# Increasing the Genetic Diagnosis Yield in Inherited Retinal Dystrophies: Assigning Pathogenicity to Novel Non-canonical Splice Site Variants

**DOI:** 10.3390/genes11040378

**Published:** 2020-03-31

**Authors:** Vasileios Toulis, Vianney Cortés-González, Marta de Castro-Miró, Juliana Ferraz Sallum, Jaume Català-Mora, Cristina Villanueva-Mendoza, Marcela Ciccioli, Roser Gonzàlez-Duarte, Rebeca Valero, Gemma Marfany

**Affiliations:** 1 DBGen Ocular Genomics, Barcelona 08011, Spain; vtoulis@ub.edu (V.T.); vianney.cortes@hotmail.com (V.C.-G.); mart.dmc@gmail.com (M.d.C.-M.); rgonzalez@ub.edu (R.G.-D.); 2 Departament de Genètica, Microbiologia i Estadística, Facultat de Biologia, Universitat de Barcelona, Avda. Diagonal 643, Barcelona 08028, Spain; 3 Departamento de Genética, Asociación para Evitar la Ceguera en México, Ciudad de México 04030, Mexico; villanuevacristina@hotmail.com; 4 Instituto de Genética Ocular and Ophthalmology Department Federal University of São Paulo (UNIFESP), São Paulo 04552-050, Brazil; juliana@pobox.com; 5 Hospital Sant Joan de Deu, Esplugues de Llobregat, Barcelona 08950, Spain; jcatalam@sjdhospitalbarcelona.org; 6 Stargardt APNES-Retina, Buenos Aires, Argentina; marcelaciccioli@yahoo.com.ar; 7 Centro de Investigación Biomédica en Red de Enfermedades Raras (CIBERER), Instituto de Salud Carlos III, Barcelona 08028, Spain; 8 IBUB-IRSJD, Barcelona 08028, Spain

**Keywords:** inherited retinal dystrophies, non-canonical splice sites, aberrant splicing, minigenes

## Abstract

Aims: We aimed to validate the pathogenicity of genetic variants identified in inherited retinal dystrophy (IRD) patients, which were located in non-canonical splice sites (NCSS). Methods: After next generation sequencing (NGS) analysis (target gene panels or whole exome sequencing (WES)), NCSS variants were prioritized according to in silico predictions. In vivo and in vitro functional tests were used to validate their pathogenicity. Results: Four novel NCSS variants have been identified. They are located in intron 33 and 34 of *ABCA4* (c.4774-9G>A and c.4849-8C>G, respectively), intron 2 of *POC1B* (c.101-3T>G) and intron 3 of *RP2* (c.884-14G>A). Functional analysis detected different aberrant splicing events, including intron retention, exon skipping and intronic nucleotide addition, whose molecular effect was either the disruption or the elongation of the open reading frame of the corresponding gene. Conclusions: Our data increase the genetic diagnostic yield of IRD patients and expand the landscape of pathogenic variants, which will have an impact on the genotype–phenotype correlations and allow patients to opt for the emerging gene and cell therapies.

## 1. Introduction

Inherited retinal dystrophies (IRDs) constitute a group of clinically and genetically heterogenous Mendelian disorders that lead to irreversible and progressive visual impairment due to dysfunction or loss of photoreceptors. During the past two decades, over 250 IRD causative genes have been described (https://sph.uth.edu/retnet/), most of them using the conventional capillary-based DNA Sanger sequencing methods. Of late, rapid, efficient and cost-effective next-generation sequencing (NGS) has revolutionized the genetic diagnosis field, raising the genetic yield to over 70% when including coding and flanking non-coding regions [1,2]. However, NGS approaches highlight a large number of genetic variants that in the absence of a clear-cut causative mutation have to be prioritized—a non-trivial task. This is particularly evident when the identified genetic variants are located outside canonical splice sites or embedded in deep-intronic regions. In these cases, in silico predictions provide relevant clues that require in vitro or in vivo functional assays to validate their pathogenicity. 

To break the current ceiling in the genetic diagnosis of clinically heterogeneous diseases (such as IRDs) and to determine genotype–phenotype correlations, novel protocols for the analysis of copy number variants, and the identification of mutations that either cause aberrant splicing products or impact in the regulation of gene expression, have to be developed and implemented in routine diagnostic assays [3,4,5].

In this context, current estimates indicate that at least 33% of disease-causing mutations alter pre-mRNA splicing [6], which is most probably an underestimation because: (i) NGS analysis (WES and gene panels) are mostly restrained to genetic variants located at the boundaries of canonical splice-site sequences; (ii) some synonymous substitutions, in fact, disrupt splicing regulatory elements such as ESEs/ISE (exonic/intronic splicing enhancers) and ESSs/ISSs (exonic/intronic splicing silencers) [7,8]; and (iii) genetic variants mapping at either non-canonical splice sites (NCSS) or hidden within large introns go mostly undetected. 

Our work and that from several groups show that functional validation of variants causing aberrant splicing is attainable in conventional diagnostic centers and amenable to be incorporated in routine diagnostic protocols [3,9,10,11]. Here we present the functional characterization of new NCSS mutations: two located in intron 33 and 34 of the *ABCA4* gene, one located in intron 2 of *POC1B* gene, and one located in intron 3 of *RP2* gene. Our findings expand the universe of pathogenic mutations located in non-coding sequences and highlight the relevance of paying specific attention to NCSS genetic variants. The validation of their phenotypic impact secures genetic diagnosis, with clear benefit for the patient and the clinician, and opens the way to devise personalized gene-therapy strategies based on antisense-oligonucleotides.

## 2. Materials and Methods 

### 2.1. Clinical Diagnosis

Patients enrolled in this study were clinically diagnosed with either Retinitis Pigmentosa, Stargardt disease or cone-rod dystrophy on the basis of ophthalmic studies that included visual acuity and visual field tests, fundus ophthalmoscopy, optical coherence tomography (OCT) and electroretinographic (ERG) studies (Table 1).

### 2.2. Samples

After approval from the Bioethics Committee of the Universitat de Barcelona (Institutional Review Board IRB_00003099, 2016), written informed consents for genetic testing were obtained from patients and families, following the recommendations of the American College of Medical Genetics (ACMG) and abiding to the tenets of the Declaration of Helsinki, prior to donation of blood samples. Peripheral blood DNA samples from patients and available relatives were obtained using the QIAamp DNA Blood Maxi Kit (Qiagen, Hilden, Germany). Genomic DNA from probands was analyzed by targeted gene panel sequencing. The targeted gene panel comprised the coding regions of 346 genes and 65 intronic sequences, which include all IRD genes plus genes causing other visual disorders (the complete list of genes is available at www.dbgen.com). Variants identified in genes associated with retinal disorders were carefully selected by the predicted molecular phenotypic effect of the clinical disorder as well as allele frequencies in gnomAD and in our control cohort. Candidate variants were validated by Sanger sequencing and confirmed by cosegregation analysis (Table 2).

### 2.3. In Silico Analysis of the Variant Effects on Splicing 

The potential effect of the identified non-canonical splice variants (c.884-14G>A in *RP2*, c.4774-9G>A and c.4849-8C>G in *ABCA4*, and c.101-3T>G, in *POC1B*) on splicing was assessed comparing wild-type and mutant sequences using four different algorithms (i.e., Human SpliceSite Finder, MaxEntScan, NetGene and NNSPLICE) (Table 3).

### 2.4. In Vivo Splicing Analysis of RP2 

After previous confirmation that *RP2* is expressed in blood, samples from patient and control were used to analyze the splicing pattern of these genes. Cycloheximide and RNAlater solution (ThermoFisher Scientific, Waltham, MA, USA) were added as described elsewhere [9]. Total RNA was extracted using RiboPure-Blood Kit (Life Technologies, now ThermoFisher Scientific, Waltham, MA, USA) according to the manufacturer’s instructions. cDNA strands were synthesized using the qScript cDNA Synthesis Kit (Qiagen, Hilden, Germany). To analyze the effect of the identified variants in splicing events, first-strand cDNA templates were used to amplify the region of interest from exon 2 to 5 of *RP2* with specific primers (*RP2* Exon2F: 5′-GACAGAAGAGCAGCGATGAAT-3′, *RP2* Exon5R: 5′CATATTCCCATCTGTATATCAGC-3′). PCR reactions were performed as previously described [12], and the products were analyzed by Sanger sequencing.

### 2.5. In Vitro Splicing Assays of ABCA4 and POC1B in HEK293T Cells

For individual analysis of each *ABCA4* variant (c.4774-9G>A and c.4849-8C>G), fragments of 608 bp that spanned part of exon 33 and exons 34-35 (using specific primers Exon33F: 5′-ATGGAGGAATTTCCATTGGAGG-3′ and Exon35R: 5′-AAGATGGCGTTGTGGGCCAC-3′) were amplified from the patient’s genomic DNA. For individual analysis of *POC1B* variant (c.101-3T>G), one genomic fragment of 533 bp flanking exon 3 (using specific primers *POC1B* 2inFw 5′-CCTGCTGTGTGCTTACATAGG-3′ and *POC1B* 3inRv-5´-TGACTTTACCACAAGGGCAGC-3´) were also amplified from patient and control.

Amplified fragments were subcloned into the HIV *tat* intron of the pSPL3 expression vector (Addgene, Watertown, MA, USA). HEK293T cells were seeded on 24-well plates (50,000 cells/well) and grown in DMEM (Thermofisher Scientific, Waltham, MA, USA) supplemented with 10% of FBS (fetal bovine serum). After 24 h, cells were lipofected with constructs bearing either the pSPL3-ABCA4 WT minigene, pSPL3-ABCA4 MUT minigenes (containing the c.4774-9G>A or c.4849-8C>G variants), pSPL3-POC1B WT minigene, pSPL3-POC1B MUT minigene (containing the c.101-3T>G variant), or the empty pSPL3 vector. Transfections cycloheximide treatment, RNA isolation, reverse-transcription PCR, and Sanger sequencing of amplified cDNA bands were performed as described elsewhere [9].

## 3. Results

Several patients affected with retinitis pigmentosa, retinitis pigmentosa with macular affectation, and cone-rod dystrophy that were referred by their clinicians requested genetic diagnosis. Clinical tests including visual acuity and visual field tests, fundus ophthalmoscopy, optical coherence tomography (OCT) and electroretinogram (ERG) studies had been previously performed (Table 1). 

**Figure 1:**
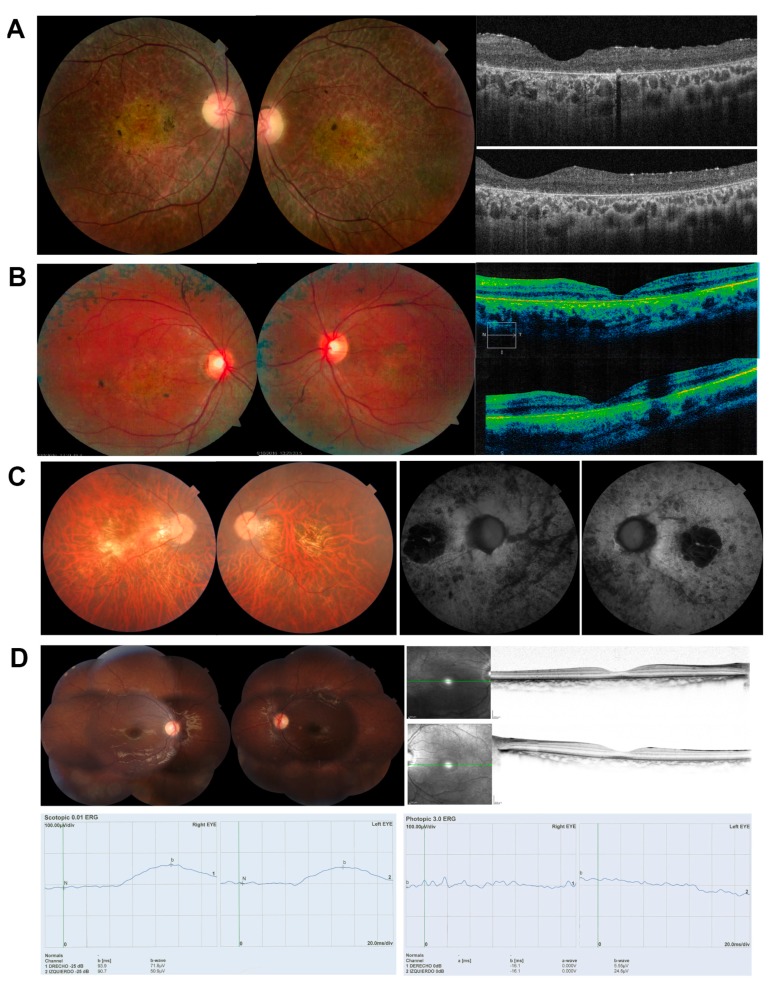
Ocular phenotypes of patients analyzed in this study, with fundus examination, autofluorescence imaging, OCT imaging and ERGs (if available). (**A**) DBG1, clinically diagnosed of RP; Fundus image showing retinal vascular attenuation, central EPR and retinal atrophy, with peripheral retinal bony spicule hyperpigmentation. OCT shows diffuse retinal thinning, and severe alteration of the external layers. (**B**) DBG2, clinically diagnosed of RP with macular affectation; Fundus showing the central macular area exhibiting hypoautofluorescence corresponding to foveal area. Macular area of granular aspect with points of hyperautofluorescence. There are several scattered parches of hypoautofluorescence located out of vascular arcades at temporal and nasal side; OCT shows preserved foveal morphology and thinning of the macula in both eyes. (**C**) DBG3, clinically diagnosed of RP; Fundus showing tilted disc, peripapilary atrophy, atrophic RPE at the macula with a geographical shape, some vitreous opacities and some bony spicule pigmentation at the fundus equator. The atrophic macular area presents hypoautofluorescence with a small sparing area at the fovea. (**D**) DBG4 clinically diagnosed of Cone-Rod Dystrophy. Both eyes with retinal pigmentary changes and mild central hyperautofluorescence, with normal OCT; ERGs with very reduced scototpic (left) and photopic (right) response.

Patient DBG1 showed typical RP disease clinical traits, retinal vascular attenuation, EPR with retinal atrophy and peripheral retinal bony spicule hyperpigmentation (Figure 1A, Table 1). Patient DBG2, who was initially referred as RP with macular affectation, showed early onset photophobia, decreased central vision, peripheral retinal lesions with diffuse chorioretinal atrophy (Figure 1B, Table 1). Patient DBG3 suffered from night blindness and high myopia since childhood and his fundus examination showed peripapillary atrophy, atrophic Retinal Pigment Epithelium (RPE) at the macula, and vascular attenuation, and was initially clinically diagnosed as RP (Figure 1C, Table 1). Finally, patient DBG4 complained of visual acuity impairment and photophobia since childhood. Fundoscopy showed pigmentary changes and electroretinogram demonstrated a reduced photopic response. He was clinically diagnosed as being affected of cone-rod dystrophy (Figure 1D, Table 1). The propositus’ 10 year-old-brother had similar phenotypic characteristics with tapetal-like sheen.

We addressed the genetic diagnosis of these patients using target gene panel NGS. In patients DBG1 and DBG2 (initially diagnosed of RP), we detected only one previously reported *ABCA4* pathogenic allele in each, namely, c.735T>G p.Tyr245* and c.2894A>G p.Asn965Ser. *ABCA4* was a good candidate gene to explain the phenotype presented by the patients, but the second allele was missing. On the other hand, in patients DBG3 and DBG4, no clear pathogenic allele in the coding region of any IRD was identified. A careful examination of NGS data showed that all these patients carried intronic variants mapping close to the intron–exon boundaries (NCSS) in candidate genes (*ABCA4, RP2* and *POC1B*) (Table 2). Cosegregation analysis confirmed biallelic inheritance of *ABCA4* variants in DBG2, biallelic inheritance of *POC1B* variants in patient DBG4, and hemizygosity of the *RP2* variant in patient DBG3 (Table 2). Concerning DBG1, an affected sister shared the same genotype in *ABCA4* than the proband, supporting that this new NCSS variant is the second pathogenic allele. Nonetheless, as the progenitors were not available, we could not confirm biallelism in trans by cosegregation analysis. The very low frequency of these alleles and the absence of homozygotes in normal population databases (GnomAD) supported that these NCSS variants might be causative of aberrant splicing events. In silico prediction analyses using HSF, MaxEntScan, NetGene, and NNSplice programs also confirmed their putative pathogenic molecular effect (Table 3). In all cases, the score value of the reported acceptor or donor splice site was lower in the NCSS variant allele. Furthermore, for variants *ABCA4* c.4849-8C>G, *ABCA4* c.4774-9G>A, *RP2* c.884-14G>A and *POC1B* c.101-3T>G, a new acceptor splice site was generated that displayed a higher score value, highly indicative of the generation of new splice sites that could interfere with the normal splicing events.

These results prompted us to validate the effect on each of these variants on the splicing pattern of the corresponding gene transcripts either in vivo (*RP2*, which is expressed in blood) or in vitro (*ABCA4* and *POC1B* minigenes transfected in cultured cell lines), following established methodologies [3,9,11,12]. 

**Figure 2:**
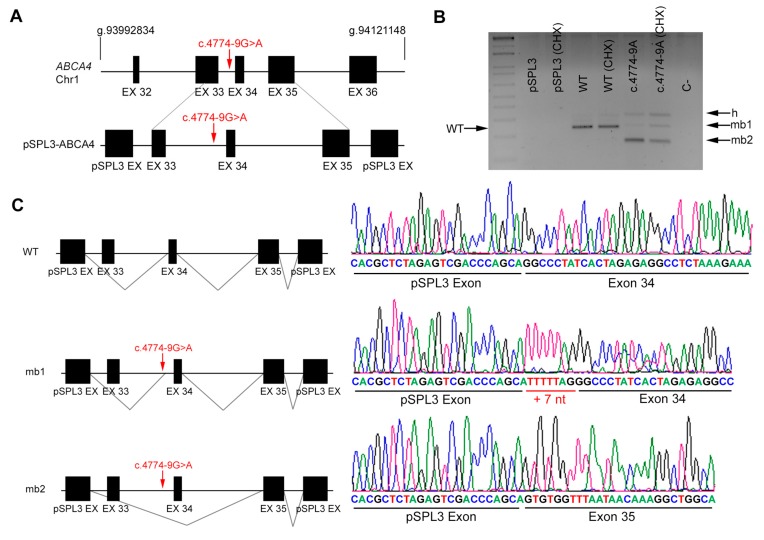
In vitro splicing analysis of the non-canonical splice site (NCSS) variant identified in *ABCA4* in patient DBG1. (**A**) Genomic position of the identified NCSS variant in intron 33 of the *ABCA4* gene (c.4774 -9G>A). Diagram showing the genomic region amplified from patient’s DNA, cloned into the pSPL3 vector. Note that exon 33 was not included in full. (**B**) Analysis of *ABCA4* mRNAs from HEK293T cells transfected with either empty vector, WT or mutant genomic sequences (treated or untreated with cycloheximide, CHX). The band of 247 bp corresponds to the wild-type transcript (WT), whereas cells transfected with pSPL3 carrying the NCSS variant produced two different aberrant transcripts (mb1 and mb2). h indicates the heteroduplex band of mb1 and mb2. C- indicates the PCR negative control. (**C**) Subsequent Sanger sequencing of cloned individual bands (indicated in the left diagrams) and comparison to the wild-type transcript confirmed the insertion of 7 bp from intron 33 (mb1) due to acceptor splice site shift as well as exon skipping of exon 34 (mb2).

**Figure 3:**
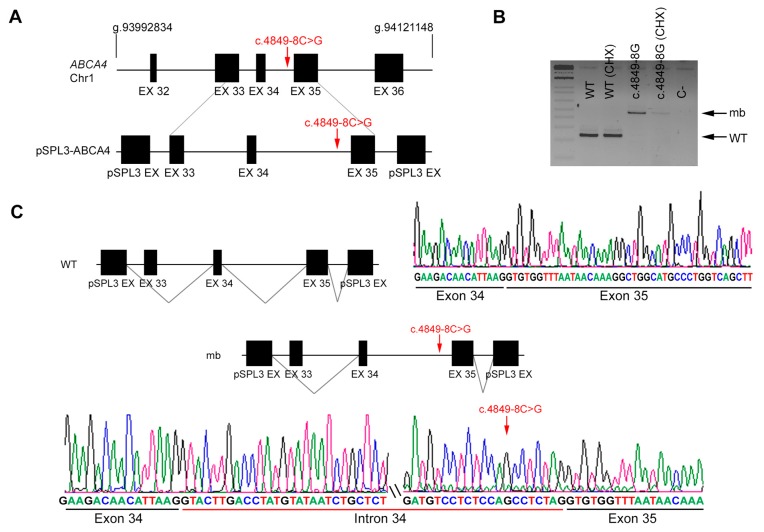
In vitro splicing analysis of the non-canonical splice site (NCSS) variant identified in *ABCA4* in patient DBG2. (**A**) Genomic position of the identified NCSS variant in intron 34 of the *ABCA4* gene (c.4849-8C>G). Diagram showing the genomic region amplified from patient’s DNA, cloned into the pSPL3 vector. Note that exon 33 was not included in full. (**B**) Analysis of *ABCA4* mRNAs from HEK293T cells transfected with either WT or mutant genomic sequences (treated or untreated with cycloheximide, CHX). The band of 247 bp corresponds to the wild-type transcript (WT), whereas cells transfected with pSPL3 carrying the NCSS variant produced one aberrant transcript of 477 bp (indicated as mb). C- indicates the PCR negative control. (**C**) Subsequent Sanger sequencing of cloned individual bands confirmed intron 34 retention due to the NCSS variant.

Since *ABCA4* is not expressed in tissues other than the retina, we generated constructs that carried identified variants within their genomic context from patient and control DNA. The corresponding amplified genomic regions were cloned into the pSPL3 backbone, a vector designed for in vitro splicing assays. Cells were transfected with the wild-type and mutant sequences, and cycloheximide was added in order to prevent nonsense-mediated decay of any aberrant transcripts, which could otherwise go undetected [9]. Transcripts produced from each construct in treated and untreated cells were purified and sequenced to compare the effect of the variants. Variant c.4774-9G>A, which potentially introduced a new AS in intron 33 (Figure 2A), clearly produced two aberrant transcripts, one with 7 additional nucleotides from intron 33 (+ 7 nt) due to shifting of the AS, and another with skipping of exon 34 (Figure 2B, mb1 and mb2, respectively). Both transcripts produce a frameshift and the introduction of premature truncating STOP codons (Figure 2C). On the other hand, variant c.4849-8C>G, which lowers the value of the polypyrimidine tract of the AS in intron 34, produces transcripts with intron 34 retention, which would also result in premature protein truncation (Figure 3).

**Figure 4:**
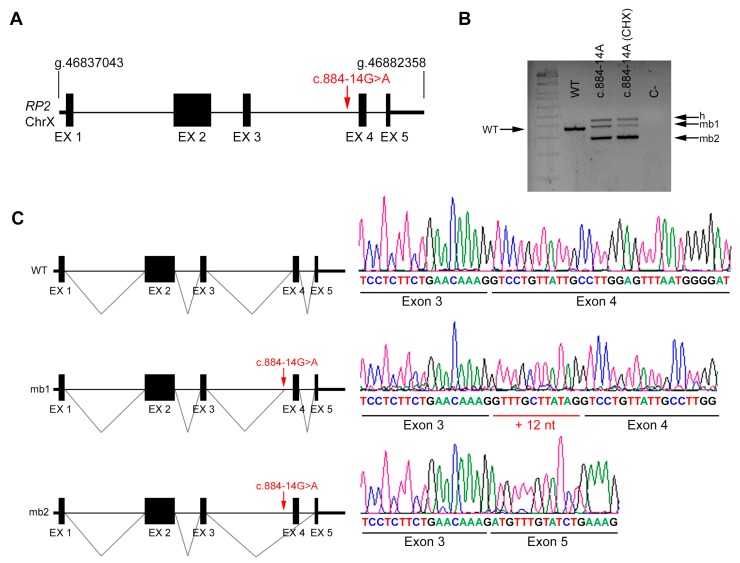
In vivo splicing analysis of the non-canonical splice site (NCSS) variant identified in *RP2*. (**A**) Diagram showing the genomic position of the NCSS variant in intron 3 of the *RP2* gene in chromosome X (c.884 -14G>A) from patient R1. (**B**) In vivo analysis of *RP2* mRNAs from control (WT) and patient’s blood samples (treated or untreated with cycloheximide, CHX). The band of 370 bp corresponds to the wild-type transcript (WT), whereas blood from the hemizygous patient carrying the NCSS variant resulted into two different aberrant transcripts (mb1 and mb2). h indicates the heteroduplex band of mb1 and mb2. C- indicates the PCR negative control. (**C**) Subsequent Sanger sequencing of cloned individual bands, indicated in the left diagrams, confirmed the in-frame insertion of 12 bp (mb1) and exon skipping of exon 4 (mb2) compared to the wild-type transcript.

*RP2* is an X-linked gene that encodes a ciliary protein that is widely expressed in many cells and tissues, among them blood. In vivo analysis of the *RP2* transcripts in a patient’s fresh blood sample was performed and compared to a control (Figure 4A). Variant c.884-14G>A lowers the score value of the intron 3 AS of *RP2* and generates a new in-frame AS. Our results support the pathogenicity of this variant since two aberrant transcripts are produced in vivo; one shows the addition of 12 nt between exon 3 and 4, and the other shows exon 4 skipping (Figure 4B,C). This transcript is out-of-frame and introduces a premature STOP codon.

**Figure 5:**
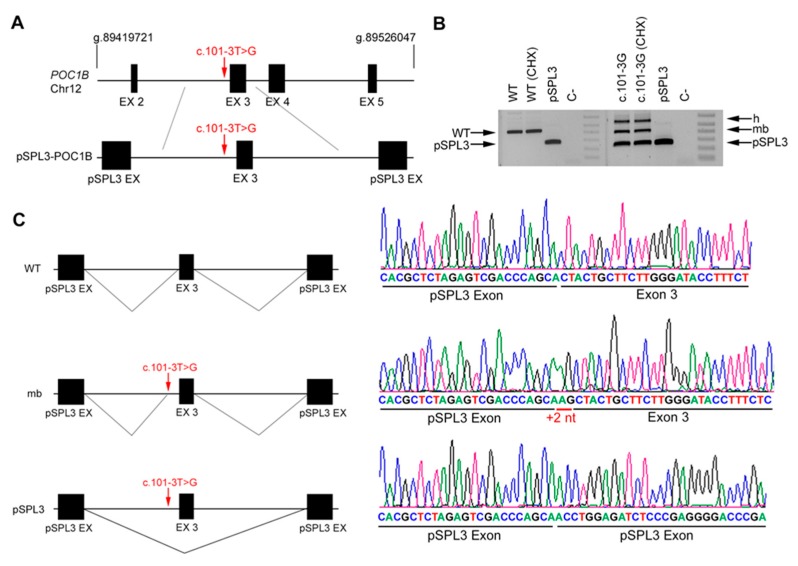
In vitro splicing analysis of the non-canonical splice site (NCSS) variant identified in *POC1B*. (**A**) Genomic position of the NCSS variant in intron 2 of the *POC1B* gene (c.101-3T>G) from patient DBG4. Diagram showing the genomic region between introns 2 and 3 amplified from patient’s DNA, cloned into the pSPL3 vector. (**B**) Analysis of *POC1B* mRNAs from HEK293T cells transfected with either empty vector, WT or mutant genomic sequences (treated or untreated with cycloheximide, CHX). The band of 429 bp corresponds to the wild-type transcript (WT), whereas cells transfected with pSPL3 carrying the NCSS variant produced one aberrant transcript (mb) plus the same transcript band produced by the pSPL3 control vector, indicating an exon skipping event. h indicates the heteroduplex band of the mb and skipped transcript products. C- indicates the PCR negative control. (**C**) Subsequent Sanger sequencing of cloned individual bands, indicated in the left diagrams, confirmed the insertion of +2 nt (mb) and the skipping of exon 2 compared to the wild-type transcript.

The effect of *POC1B* variant was approached in vitro. Variant c.101-3T>G, which alters the AS of intron 2, produced only two aberrant transcripts: one that included two extra nucleotides at the 5′ of exon 3, and the other showing skipping of exon 3 (Figure 5). These two aberrant transcripts are out-of-frame and would cause a premature truncation of the protein. 

## 4. Discussion

One of the current challenges in genetic diagnosis is the identification of mutations located in non-coding sequences, e.g. mutations in regulatory and intronic regions [13]. WES and target gene panels, which are the tools of choice for routine genetic diagnoses, mainly capture exonic and exon–intron boundary sequences, and also the filtering bioinformatics algorithms used for analysis mostly focus on variants that alter the coding-sequence or the consensus DS and AS splicing sites [14,15]. Without the addition of deep-intronic and NCSS variants, only around 50% of the cases in IRDs are diagnosed conclusively. The rest of the cases remain unsolved, with either the identification of a single pathogenic allele or even none. A step further in genetic diagnosis has been attained when deep-intronic variants that alter the splicing pattern have been identified, as is the case in Stargardt disease, a macular disorder with a very well defined clinical phenotype and a major causative gene, *ABCA4*. In fact, the introduction of the whole *ABCA4* locus in the target NGS panels clearly helps to increase the genetic yield in Stargardt disease patients [16,17]. 

Potential pathogenic nucleotide variants that are overlooked in the WES and target gene sequencing algorithms for variant prioritization are located in non-canonical splice sites (NCSS). These variants do not directly affect the primary sequence of the consensus donor and acceptor sites, but they can instead disrupt or alter the splicing motif recognition by the spliceosome and adjuvant factors, for instance, by shifting the percentage of pyrimidines in the polypyrimidine tract flanking the AS or by disrupting conserved Exon Splicing Enhancers (ESEs). Not all variants mapping at close locations of consensus splice sites will be have an effect on splicing. In silico predictions provide score values to splice-site motifs in the sequence carrying the identified NCSS variant and allow the comparison to the wild-type sequence. Alterations in these score values are a first clue, but indeed, functional in vitro and in vivo assays should be performed to validate the impact of these novel variants in the splicing of transcripts before they could be assigned as pathogenic. 

Confirmation of the splicing altering effect of the identified variants can be directly performed in patients by transcript analysis in blood, saliva, hair or biopsies, when the gene is expressed in these tissues [12,18], as happens with genes that encode ciliary proteins, such as *RP2* (Figure 3). However, many IRD genes are expressed only in the retina (e.g., *ABCA4*) or show a tissue-specific splicing pattern (e.g., *RPGR*) [19]. The construction of midigenes or minigenes spanning the genomic context for in vitro expression in cell cultures allows to assay the impact of the identified variant in the splicing of transcripts and thus validate pathogenicity [3]. Tissue-specific spliceosome factors might be required to observe the pathogenic effect of a particular variant. In these cases, the differentiation of patient’s iPSCs into retinal organoids mimics a physiological retinal-like setting that has been used to test mutations altering retinal-restricted splicing events [20]. Indeed, retinal organoids are very informative tools for variant validation, but this approach is costly and available to very few laboratories.

All the variants identified in this work alter the NCSS of the AS. According to our results, in one of the *ABCA4* alleles, the variant perturbs the polypyrimidine tract and as a consequence, the intron is retained in most splicing events. The other three identified alleles (in *RP2*, *POC1B* and one in *ABCA4*) generate a new consensus AS, sometimes with a stronger score value than the wild-type AS. In these cases, two different splicing effects are observed: exon skipping and the production of an aberrant transcript that adds several nucleotides to the exon, usually out-of frame. These two alterations, elongation of the upstream sequence of an exon and exon skipping, have been reported as expectable outcomes for NCSS variants [3,9,12,21]. It is difficult to assess whether any wild-type spliced transcripts are still produced in the patient’s retina, although NCSS mutations should not be expected to be as severe as mutations disrupting the consensus splice motifs. In this context, most NCSS variants might be considered as hypomorphic alleles. 

Several authors have proposed that some “missing heritability” in IRDs is due to deep-intronic and NCSS mutations [2,22,23]. At least for IRDs, *ABCA4* is the paradigm gene for intronic mutations that alter splicing. Most of the variants are novel, indicating that many mutations in this gene are private. Our results further support that some of these “hidden” IRD alleles are located in NCSS, expand the NCSS list of mutations in *ABCA4* and also bring two new genes, *RP2* and *POC1B*, to the fore. We surmise that as more data is being gathered and analyzed, more deep-intronic and non-canonical splice site mutations will come to light [9], not only in IRD genes but also in other Mendelian diseases. Considering that among the most recently reported successful gene therapies for visual disorders, antisense oligonucleotides (AONs) are particularly promising to modulate the effect of splicing mutations [20,21,22,23,24,25], their characterization becomes crucial for patients carrying these variants in order to access these emerging therapies.

## 5. Conclusions

The identification of four novels NCSS variants in *ABCA4*, *RP2*, and *POC1B* highlights the relevance of pathogenic hidden variants that alter splicing to increase the genetic diagnostic yield of IRDs. These findings contribute to define the genotype-phenotype correlations and help patients and clinicians to make the best decision in front of the emerging gene and cell therapies. 

## Figures and Tables

**Table 1:** Clinical data.

Patient	Disease	Age	A.O.	Sex	VA	BCVA	ERG
		years	years	F/M	RE/LE	RE/LE	
**DBG1**	RP	31	12	F	CF OU (counting fingers)	CF OU	Abolished photopic and scotopic.
**DBG2**	RP + macula	22	7	M	20–320/ 20–200	20–200	No data
**DBG3**	RP	40	15	M	Counting fingers	20–400	Abolished rod ERG and 88% reduced ERG in cones.
**DBG4**	CRD	8	3	M	OU: 20-100	OD: 20–40 OS: 20–50	Reduced photopic and flicker amplitude.

VA: Visual Acuity. BCAV: Best Corrected Visual Acuity; RP. Retinitis pigmentosa; CRD: Cone-rod dystrophy

**Table 2:** Genotype of IRD patients and siblings. Gene, nucleotide and exon/intron position are indicated.

			Allele 1		Allele 2	
Patient ID		Gene	Exon/Intron	Nucleotide Change	Exon/Intron	Nucleotide Change
**DBG1**	Index	*ABCA4*	6	c.735T>G	IVS33	c.4774-9 G>A
	Affected sister			c.735T>G		c.4774-9 G>A
**DBG2**	Index	*ABCA4*	19	c.2894A>G	IVS34	c.4849-8 C>G
	Unaffected mother			Reference sequence (WT)		c.4849-8 C>G
**DBG3**	Index	*RP2* (X chrom.)	IVS3	c.884-14G>A		NA
	Unaffected mother			c.884-14G>A		Reference Sequence (WT)
**DBG4**	Index	*POC1B*	IVS2	c.101-3T>G	IVS11	c.1332+5G>A
	Unaffected father			c.101-3T>G		Reference Sequence (WT)
	Unaffected mother			Reference Sequence (WT)		c.1332+5G>A
	Affected brother			c.101-3T>G		c.1332+5G>A

NA—Not applicable since patient is hemizygous for the *RP2* mutation. WT—wild-type sequence.

**Table 3:** In silico analysis of the identified variants altering Non-Canonical Splice Sites (NCSS), Gene, Location and Minimum Allele Frequency (MAF) of each variant is indicated. Relevant changes are highlighted in red.

Subject	Gene	Nucleotide Variant	Intron	MAF	HSF			MaxEntScan			NetGene		NNSplice		
					WT	MUT		WT	MUT		WT	MUT	WT	MUT	
**DBG1**	*ABCA4*	c.4774-9 G>A	IVS33	ND	AS 79.18	AS 79.0	NewAS 79.05	AS 8.06	AS 3.04	NewAS 6.08	AS 0.15	AS 0.15	AS 0.77	AS NR	NewAS 0.93
**DBG2**	*ABCA4*	c.4849-8C>G	IVS34	ND	AS 86.55	AS 84.0	NewAS 89.73	AS 10.9	AS 4.46	NewAS 3.64	AS NR	AS NR	AS 0.98	AS **0.91**	
**DBG3**	*RP2*	c.884-14G>A	IVS3	ND	AS 85.41	AS NR	NewAS 78.72	AS 8.69	AS **5.5**		AS 0.90	AS **0.87**	AS 0.95	AS **0.92**	NewAS 0.66
**DBG4**	*POC1B*	c.101-3T>G	IVS2	0.0004	AS 79.56	AS 76.9	NewAS 79.84	AS 7.64	AS −1.4		AS 0.15	AS NR	AS 0.94	AS NR	

ND: No data available in GnomAD. AS: Acceptor site. DS: Donor site. NR: Not recognized. NewAS: New acceptor site.

## References

[1] Ellingford J.M., Barton S., Bhaskar S., O’Sullivan J., Williams S.G., Lamb J.A., Panda B., Sergouniotis P.I., Gillespie R.L., Daiger S.P. (2016). Molecular findings from 537 individuals with inherited retinal disease. J. Med. Genet..

[2] Zernant J., Xie Y.A., Ayuso C., Riveiro-Alvarez R., Lopez-Martinez M.-A., Simonelli F., Testa F., Gorin M.B., Strom S.P., Bertelsen M. (2014). Analysis of the *ABCA4* genomic locus in Stargardt disease. Hum. Mol. Genet..

[3] Sangermano R., Khan M., Cornelis S.S., Richelle V., Albert S., Garanto A., Elmelik D., Qamar R., Lugtenberg D., van den Born L.I. (2018). *ABCA4* midigenes reveal the full splice spectrum of all reported noncanonical splice site variants in Stargardt disease. Genome Res..

[4] Carss K.J., Arno G., Erwood M., Stephens J., Sanchis-Juan A., Hull S., Megy K., Grozeva D., Dewhurst E., Malka S. (2017). Comprehensive rare variant analysis via whole-genome sequencing to determine the molecular pathology of inherited retinal disease. Am. J. Hum. Genet..

[5] Simunovic M.P., Jolly J.K., Xue K., Edwards T.L., Groppe M., Downes S.M., MacLaren R.E. (2016). The spectrum of CHM gene mutations in choroideremia and their relationship to clinical phenotype. Investig. Opthalmol. Vis. Sci..

[6] Sibley C.R., Blazquez L., Ule J. (2016). Lessons from non-canonical splicing. Nat. Rev. Genet..

[7] Kataoka N. (2017). Modulation of aberrant splicing in human RNA diseases by chemical compounds. Hum. Genet..

[8] Zhu Y., Deng H., Chen X., Li H., Yang C., Li S., Pan X., Tian S., Feng S., Tan X. (2019). Skipping of an exon with a nonsense mutation in the *DMD* gene is induced by the conversion of a splicing enhancer to a splicing silencer. Hum. Genet..

[9] Valero R., de Castro-Miró M., Jiménez-Ochoa S., Rodríguez-Ezcurra J.J., Marfany G., Gonzàlez-Duarte R. (2019). Aberrant splicing events associated to *CDH23* noncanonical splice site mutations in a proband with atypical Usher syndrome 1. Genes.

[10] Liquori A., Vaché C., Baux D., Blanchet C., Hamel C., Malcolm S., Koenig M., Claustres M., Roux A.-F. (2016). Whole *USH2A* gene sequencing identifies several new deep intronic mutations. Hum. Mutat..

[11] Jaijo T., Aller E., Aparisi M., García-García G., Hernan I., Gamundi M., Nájera C., Carballo M., Millán J. (2011). Functional analysis of splicing mutations in *MYO7A* and *USH2A* genes. Clin. Genet..

[12] Pomares E., Riera M., Castro-Navarro J., Andrés-Gutiérrez Á., Gonzàlez-Duarte R., Marfany G. (2009). Identification of an intronic single-point mutation in *RP2* as the cause of semidominant X-linked retinitis pigmentosa. Investig. Opthalmol. Vis. Sci..

[13] Zernant J., Lee W., Nagasaki T., Collison F.T., Fishman G.A., Bertelsen M., Rosenberg T., Gouras P., Tsang S.H., Allikmets R. (2018). Extremely hypomorphic and severe deep intronic variants in the *ABCA4* locus result in varying Stargardt disease phenotypes. Mol. Case Stud..

[14] Vaz-Drago R., Custódio N., Carmo-Fonseca M. (2017). Deep intronic mutations and human disease. Hum. Genet..

[15] Lord J., Gallone G., Short P.J., McRae J.F., Ironfield H., Wynn E.H., Gerety S.S., He L., Kerr B., Johnson D.S. (2019). Pathogenicity and selective constraint on variation near splice sites. Genome Res..

[16] Runhart E.H., Valkenburg D., Cornelis S.S., Khan M., Sangermano R., Albert S., Bax N.M., Astuti G.D.N., Gilissen C., Pott J.-W.R. (2019). Late-onset Stargardt disease due to mild, deep-intronic *ABCA4* alleles. Investig. Opthalmol. Vis. Sci..

[17] Nassisi M., Mohand-Saïd S., Andrieu C., Antonio A., Condroyer C., Méjécase C., Varin J., Wohlschlegel J., Dhaenens C.-M., Sahel J.-A. (2019). Prevalence of *ABCA4* deep-intronic variants and related phenotype in an unsolved “one-hit” cohort with Stargardt disease. Int. J. Mol. Sci..

[18] Steele-Stallard H.B., Le Quesne Stabej P., Lenassi E., Luxon L.M., Claustres M., Roux A.-F., Webster A.R., Bitner-Glindzicz M. (2013). Screening for duplications, deletions and a common intronic mutation detects 35% of second mutations in patients with *USH2A* monoallelic mutations on Sanger sequencing. Orphanet J. Rare Dis..

[19] Vervoort R., Lennon A., Bird A.C., Tulloch B., Axton R., Miano M.G., Meindl A., Meitinger T., Ciccodicola A., Wright A.F. (2000). Mutational hot spot within a new *RPGR* exon in X-linked retinitis pigmentosa. Nat. Genet..

[20] Garanto A., Duijkers L., Tomkiewicz T.Z., Collin R.W.J. (2019). Antisense oligonucleotide screening to optimize the rescue of the splicing defect caused by the recurrent deep-intronic *ABCA4* variant c.4539+2001G>A in Stargardt Disease. Genes.

[21] Fadaie Z., Khan M., Del Pozo-Valero M., Cornelis S.S., Ayuso C., Cremers F.P.M., Roosing S., The ABCA Study Group (2019). Identification of splice defects due to noncanonical splice site or deep-intronic variants in ABCA4. Hum. Mutat..

[22] Bauwens M., Garanto A., Sangermano R., Naessens S., Weisschuh N., De Zaeytijd J., Khan M., Sadler F., Balikova I., Van Cauwenbergh C. (2019). *ABCA4*-associated disease as a model for missing heritability in autosomal recessive disorders: Novel noncoding splice, cis-regulatory, structural, and recurrent hypomorphic variants. Genet. Med..

[23] Sangermano R., Garanto A., Khan M., Runhart E.H., Bauwens M., Bax N.M., van den Born L.I., Khan M.I., Cornelis S.S., Verheij J.B.G.M. (2019). Deep-intronic *ABCA4* variants explain missing heritability in Stargardt disease and allow correction of splice defects by antisense oligonucleotides. Genet. Med..

[24] Garanto A., Collin R.W.J. (2018). Design and in vitro use of antisense oligonucleotides to correct pre-mRNA splicing defects in inherited retinal dystrophies. Methods Mol. Biol..

[25] Vázquez-Domínguez I., Garanto A., Collin R.W.J. (2019). Molecular therapies for inherited retinal diseases—Current standing, opportunities and challenges. Genes.

